# The importance of endometrial nerve fibers and macrophage cell count in the diagnosis of endometriosis 

**Published:** 2013-05

**Authors:** Cihan Cetin, Hasan Serdaroglu, Sitki Tuzlali

**Affiliations:** 1 *Department of Obstetrics and Gynecology, Istanbul School of Medicine, Istanbul University, Istanbul, Turkey.*; 2 *Department of Obstetrics and Gynecology, Reproductive Endocrinology and Infertility Unit, Istanbul School of Medicine, Istanbul University, Istanbul, Turkey.*; 3 *Department of Pathology, Gynecopathology Unit, Istanbul School of Medicine, Istanbul University, Istanbul, Turkey. *

**Keywords:** *Endometriosis*, *Macrophage*, *CD68 Antigen*, *Neurofilament Proteins*

## Abstract

**Background: **Endometriosis is a disease that is hard to diagnose without the gold standard method, laparoscopy. An easier diagnostic method is needed.

**Objective:** The aim of the study is to determine whether the number of macrophage cells in the endometrium and/or the detection of nerve fibers can be used in the diagnosis of endometriosis.

**Materials and Methods: **Endometrial sampling was done to 31 patients prior to laparoscopy (L/S) or laparotomy (L/T) at Istanbul University Istanbul School of Medicine Hospital between January 2010 February 2011. Also 34 patients who were retrospectively chosen from their files were added to the study. 5 patients were excluded from the study. Totally, 31 patients were placed in the endometriosis and 29 patients in the control group. Endometrial samples were evaluated immunohistochemically with the markers protein gene product 9.5 (PGP 9.5) and neurofilament (NF) for nerve fibers and CD68 for macrophages.

**Results:** None of the samples were stained with PGP 9.5 and NF. As for CD68+cells, no statistically significant difference was observed between groups (endometriosis: 216.10±104.41; control: 175.93±43.05, p=0.06). Results were also evaluated in the subgroups of menstruel phases and disease stages. Only in the proliferative phase there was a significant increase in the endometriosis group (p=0.03). No significant difference was observed between the stages.

**Conclusion:** The detection of nerve fibers in the eutopic endometrium with the markers of PGP 9.5 and NF is not found to be helpful in the diagnosis of endometriosis. Macrophage cells may be helpful in the diagnosis only in the proliferative phase.

## Introduction

Endometriosis has a prevalence of 8-10% among women in reproductive age (1). Endometriosis is a benign chronic disease defined as the presence of endometrial stroma and glands in tissues outside the uterine cavity. Although it is mostly seen in the pelvis, it can be encountered all around the body. Endometriosis can cause infertility and/or pain symptoms like dysmenorrhea, dyspareunia, dysuria, and dyschezia (2). However, it is important not to forget that it may also be asymptomatic. Unfortunately, most of the time, history and physical examination alone are not enough for the diagnosis. Establishing accurate diagnosis, depending on the symptoms is quite difficult, because patients have various clinical presentations. Irritable bowel syndrome and pelvic inflammatory disease can often be hard to differentiate. Usually, diagnosis take quite long time until laparoscopy (L/S) is performed (3). Even though transvaginal ultrasound (TVU) can be helpful in the diagnosis of ovarian endometriomas, it has no value for peritoneal disease (4). 

Magnetic resonance (MRI) imaging has value in the diagnosis of endometriomas with high specifity and sensitivity. L/S is the gold standart method for the diagnosis. Entire pelvis should be systematically evaluated, the location and severity of lesions and adhesions should be noted in detail and suspected areas should be biopsied during L/S (5). L/S is especially preferable for infertility patients with persistent and severe pain or >3 cm endometriomas, because of its diagnosing and treatment advantages. There is an inflammatory reaction in the peritoneum of endometriosis patients. This inflammation causes various cytokine production. These cytokines induce macrophage cells migrate to the eutopic endometrium causing various changes like nerve growth in the endometrium (6-9).

Macrophages are known to play an important role in the nerve growth, maturation and repair (10). It’s also known that nerve growth factor (NGF) enhances macrophage functions and plays an important role in the inflammatory response (11). These facts are the basis of the hypothesis that endometriosis patients might have nerve fibers and more macrophage cells in their endometriums.

The purpose of the study was to test the feasibility of a non-invasive diagnostic method for endometriosis. We investigated endometrial nerve fibers and macrophage cells for this purpose. Diagnosing endometriosis without an operation and with an endometrium sample that can even be taken without local anestesia in an office setting would certainly be very helpful both for patients and physicians. This procedure would save patients from the burden of an operation like L/S and cause their treatment to begin earlier.

## Materials and methods

In this cross-sectional study, 31 patients who were operated [laparoscopy (L/S) or laparotomy (L/T)] in Istanbul University Istanbul School of Medicine Hospital with various indications between January 2010 and February 2011, were prospectively assigned to the study. Preoperatively, endometrial samples were taken from these patients. In addition, 34 patients who were undergone L/S or L/T in our clinic between years of 2006-2011 were also added to the study retrospectively. 

Patients diagnosed with endometriosis confirmed by biopsy during operations were placed in the case group. If endometriosis was not diagnosed during operation, patients were placed in the control group. Exclusion criteria from the study were malignancy and adenomyosis. None of the patients were using hormone modulating medications three months prior the operation. Three of 31 patients, who were prospectively assigned to the study, were excluded from the study because of insufficient endometrial tissue for pathologic evaluation. In total 14 patients were assigned to the case group, and the other 14 to the control group. overall 6 of these patients underwent L/T and 22 patients underwent L/S.

**Figure 1:**
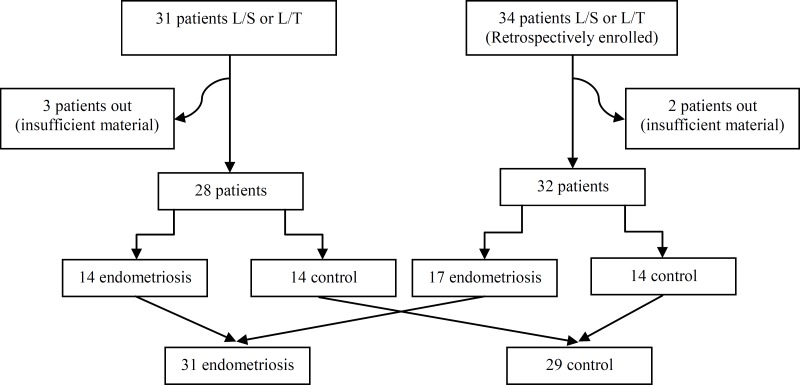
Number of patients in endometriosis and control groups (L/S: laparoscopy, L/T: laparotomy

Also, 34 patients who were randomly chosen from their files and who were previously operated in our clinic with benign indications like myoma uteri and cystoma ovarii were added to the study. Two of these patients whose endometrium samples were insufficient to evaluate under microscopy on 10 field areas were excluded from the study. Overall 4 patients had L/S, whereas 28 patients had L/T. From three patients whom endometrioma was diagnosed 17 were assigned to the case group, whereas 15 patients who were reported both in operation and pathology reports not to have endometriosis were assigned to the control group. In total 31 patients were in the endometriosis group and 29 patients were in the control group (Figure 1). Of these 60 patients, 25 patients have undergone L/S and 35 patients have undergone L/T (Table I).

Indications for operations were cystoma ovarii, infertility and endometrial hyperplasia in the endometriosis group, whereas in the control group they were cystoma ovarii, infertility, myoma uteri, chronic pelvic pain, vaginal bleeding resistant to medical treatment and tubal ligation (Table II). Before the initiation of our study, we got ethical approval from Istanbul University No: 2 Clinical Researchs Ethical Committee. All patients who willing to participate and prospectively assigned to the study have signed informed consent form.

Patients were put in litotomy position after the induction of general anestesia. Endometrial samples were taken with the help of a 4 mm thick endometrium biopsy cannula “Uterin Explora Model 1^TM^” (Coopersurgical, USA) most of the time without the need for dilatation. Endometrial sampling was done with thick biopsies from narrow endometrial areas. The immunohistochemical evaluation of a deep, colon-like endometrium sample was better than superficial, fragmented samples (2). Samples were put in 10% neutral buffered formalin solution for 18-24 hours and then embedded in paraffine blocks for pathological evaluation. Although for some patients tenaculum was needed, none of the patients had complications both during and after the procedure.

Endometriosis patients were staged according to American Fertility Society’s (AFS) endometriosis staging guideline 1996. For patients who were added to the study retrospectively, stages were determined from their previous surgery reports. All samples were evaluated by a single pathologist who was blinded to patient data and who is highly experienced in gynecopathology, using Olympus BX-51 microscope. First of all, all samples were stained with haematoxylin and eosine (H&E) and routine histologic evaluation was done. Histologic diagnoses and menstruel cycle phases were determined. The slides that showed at least ten fields of view of the tissue were included for further analyses and quantification. Instead of only functional layer, whole endometrium was examined. 

Then, immunohistochemistry was done as second stage. In our study, we used immunohistochemical markers PGP 9.5 (protein gene product 9.5) and neurofilament (NF) to detect nerve fibers. PGP 9.5 is a panneural marker both for myelinated and unmyelinated nerve fibers (Aα, Aβ, Aγ, Aδ, B, C fibers). Whereas NF is a highly specific marker for myelinated nerve fibers (Aα, Aβ, Aγ, Aδ, B fibers). 

Aδ fibers are small, myelinated fibers and these fibers are responsible for the conduction of sharp, localized pain, whereas C fibers are responsible for dull, unlocalized pain. Results were noted as there is staining for these fibers or no staining. Samples were also immunohistochemically stained with CD68 which is a specific marker for macrophages. For this purpose, CD68-KP1 clone was used. Under 400 times magnification with microscope, 10 fields were counted for macrophage cells and the results are reported as their sum.

**Immunohistochemistry**

Slides in 4 μm thickness, prepared from parafine blocks, were immunohistochemically evaluated after deparafinization procedure. Immunohistochemical procedure was done with Ventana Benchmark LT machine. Antibodies used and incubation times are listed below:

1. CD68/ Macrophage Marker Ab-3 Neomarkers, mouse antibody-Catalog # MS-397-R7 (7.0ml), ready-to-use. 16 min of incubation.
2. Neurofilament (2F11) mouse monoclonal antibody (Cell Marque), ready-to-use. One hour incubation.
3. PGP9.5 Novocastra- liquid mouse monoclonal antibody NCL-L-PGP9.5, ready-to-use. One hour incubation.

Antigen retrieval tecnique was automatically done with Benchmark LT machine, ready-to-use anticores applied and incubation periods were adjusted with positive controls. Tonsil tissue for CD68 and small intestinal tissue for NF and PGP 9.5 were used for positive controls. After counterstaining, preparates were evaluated using Olympus BX-51 microscope.

**Statistical analysis**

In order to evaluate our results, we used “Statistical Package for Social Sciences (SPSS) 16.0 for Windows” program for statistical analyses. Descriptive statistics (median, standard deviation, frequency) were used prior to detailed analyses. For comparison between groups student’s t-test and Kruskal-Wallis tests were used. Statistical significance was established at p<0.05.

## Results

Menstruel phases of the cases were categorized as histologic signs consistent with the first 14 days of the cycle as proliferative phase, 15-19 days as early secretory phase, 20-24 days as midsecretory phase and 25-28 days as late secretory phase. Some endometrium samples showed hyperestrogenic effect signs, and they were regarded as an ovulatory cycles. Although most of the cases were in the prolife rate rive phase, there was a homogeinity in the distrubution of the cases in the secretory phases. 

Patients’ ages and gravidity, parity, and abortus numbers are shown in table III. Mean ages in the endometriosis group and control group were 38.2 and 37.24 years, respectively. There was no significant difference in age, gravidity and abortus numbers among groups, but parity was significantly lower in the endometriosis group (p=0.03). Endometriosis patients, as mentioned above, were categorized as AFS 1996 guideline. 25 patients had moderate-severe (stage III-IV) endometriosis whereas 6 patients had minimal-mild (stage I-II) endometriosis. Despite long incubation times, staining with PGP 9.5 and NF which we expected to see within the stroma of the endometrium did not occur. 

But our positive control, small intestinal tissue, showed significant staining with both markers, which eliminates the argument of defective technique that could be targeted. Endometrium samples were also immunohistochemically stained with CD68 which is universally accepted as macrophage marker. With 400 times magnification under microscope, on every sample, CD68 (+) cells were counted on ten different fields and results were summed. Mean CD68 (+) cell count in the endometriosis and control groups were 216.10±104.41 and 175.93±43.05, respectively. There was no statistically significant difference between groups (p=0.06).

**Figure 2:**
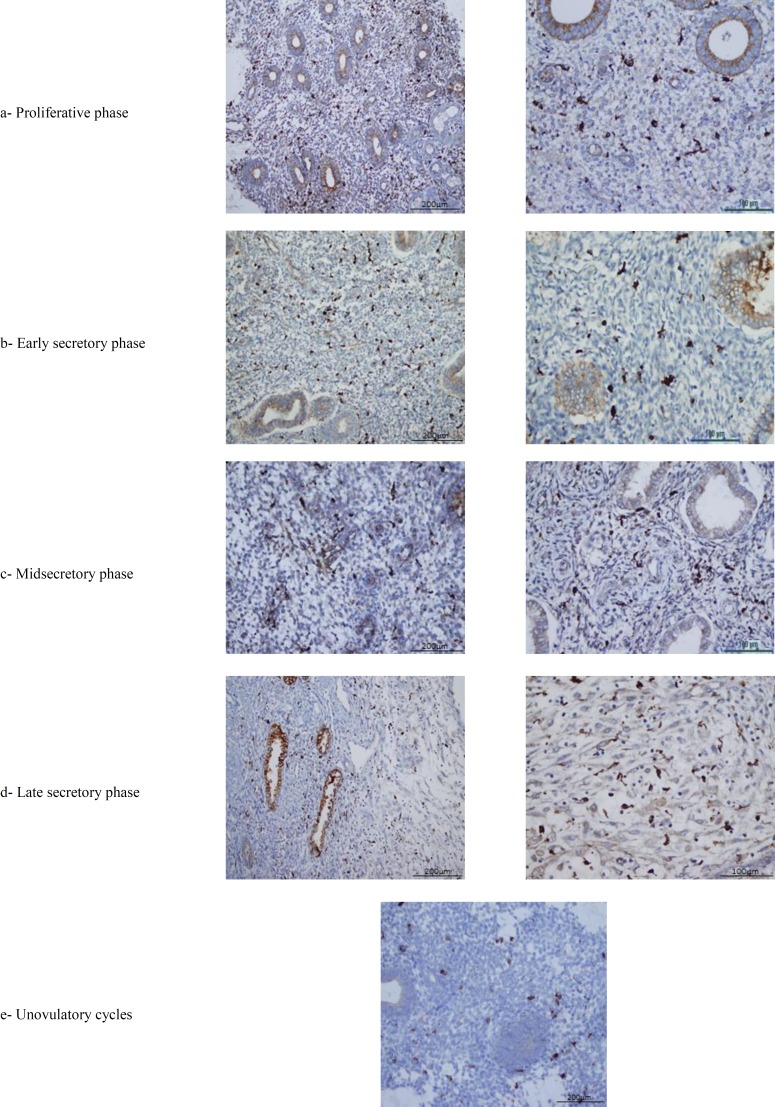
Photomicrographs taken following immunohistochemical staining for CD68 in different menstruel phases.

Menstruel cycle phase subgroups were compared with controls and only in the proliferative phase there was a statistically significant difference in the endometriosis group (p=0.03). There was not a significant difference in secretory phases and anovulatory cases subgroups compared to controls. Results are summarized in table IV. There was also, no significant difference among endometriosis stages as for CD68 (+) cell count (p=0.52) (Table IV). Photomicrographs of CD68+ cells in different menstruel cycles are shown in figure 2.

**Table I:** Operations performed

**Operation**		**N (%)**
**L/S**		25
	Diagnostic	4 (16)
	Operative	21 (84)
	LUNA	1 (5)
	Cystectomy	16 (75)
	Adhesiolysis	1 (5)
	LVAH+USO	1 (5)
	Tubal ligation	2 (10)
**L/T**		34
	Cystectomy	4 (11)
	TAH	13 (38)
	TAH+ Cystectomy	1 (2)
	TAH+USO	3 (8)
	TAH+BSO	14 (41)

L/S: laparoscopy. L/T: laparotomy. LUNA: laparoscopic uterine nerve ablation. LVAH: laparoscopy assisted vaginal hysterectomy. USO: unilateral salpingoooferectomy. BSO: bilateral salpingoooferectomy. TAH: total abdominal hysterectomy

**Table II:** Indications for operations in groups

**Indications for operations**	**Endometriosis group [n (%)]**	**Control group [n (%)]**	**Total [n (%)]**
Cystoma ovarii	29 (94)	9 (31)	38 (63)
Infertility	1 (3)	4 (14)	5 (8)
Endometrial hyperplasia	1 (3)	0	1 (2)
Chronic pelvic pain	0	2 (7)	2 (3)
Myoma uteri	0	11 (38)	11 (18)
Vaginal bleedingresistant tomedical treatment	0	1 (3)	1 (2)
Tubal ligation	0	2 (7)	2 (3)
Total	31	29	60

**Table III:** Clinical/Histological properties of patients

	**Endometriosis group Mean±SD (range)**	**Control group Mean±SD (range)**	**p value (student t-test)**
Age	38.29 ± 6.34 (26-47)	37.24 ± 5.40 (24-44)	0.49
Gravidity	1.87 ± 1.93 (0-6)	2.41 ± 1.88 (0-8)	0.27
Parity	1.19 ± 1.14 (0-3)	2.03 ± 1.64 (0-8)	0.03^*^
Abortus	0.68 ± 1.08 (0-3)	0.35 ± 0.67 (0-2)	0.15
**Histological menstruel phase**	**Endometriosis group [n (%)]**	**Control group [n (%)]**	**Total [n (** **%)]**
Proliferative	13 (42)	16 (55)	29 (48)
Early secretory	4 (13)	4 (14)	8 (13)
Midsecretory	6 (19)	2 (7)	8 (13)
Late secretory	4 (13)	5 (17)	9 (15)
Unovulatory cycles	4 (13)	2 (7)	6 (10)
Total	31	29	60
**Endometriosis stage**	**n (%)**		
I	3 (10)		
II	3 (10)		
III	9 (29)		
IV	16 (51)		
Total	31		

*Student t-test was used for comparison and (p<0.05) was accepted as statistically significant

**Table IV:** CD68 (+) cell counts in menstruel phases and in endometriosis stages

		**Endometriosis** **CD68+ cells (Mean±SD)**	**Control** **CD68+ cells (Mean±SD)**	Statistics** **(** **p-value)**
**Menstruel phase**				
	Proliferative	236.08 ± 90.57	174.81 ± 51.72	0.03^*^
	Early secretory	204.00 ± 46.10	202.00 ± 34.45	0.95
	Midsecretory	228.83 ± 183.55	178.00 ± 53.74	0.57
	Late secretory	184.50 ± 94.01	156.60 ± 12.60	0.53
	Unovulatory cycles	175.75 ± 57.77	179.00 ± 7.07	0.92
**Endometriosis stage**				
	I	154.00 ± 45.57		0.52
	II	270.00 ± 96.25		
	III	212.00 ± 90.23		
	IV	219.94 ± 120.71		

*statistically significant (p<0.05) **student t-test and Kruskal–Wallis chi-square test were used

## Discussion

In our study, the number of patients in both of the groups is similar (31 endometriosis and 29 controls). Most of the cases were in proliferative phase. There is no significant difference in age, gravidity and abortus numbers among groups, but parity is significantly low in the endometriosis group. As endometriosis has close relationship with infertility, this is an expected finding.

There are few studies in the literature that evaluates nerve fibers in the endometrium of endometriosis patients. These studies are done on human subjects and are done by Tokushige, Al-Jefout *et al* and Meibody *et al* (2, 12, 13). In all of these studies, nerve fiber detection in the eutopic endometrium was investigated immunohistochemically with nerve fiber markers NF and PGP 9.5. 

These studies found significantly high amounts of nerve fibers in endometriosis patients. In one of these studies, specifity was reported 83%, sensitivity 98%, positive predictive value (PPV) 91% and negative predictive value (NPV) 96% (2). In our study we also used PGP 9.5 and NF as markers, but we used ready-to-use markers that is different from other studies.

Another study about the endometrial nerve fibers is done by Bokor *et al* but this study does not exactly match with ours (14). That’s because, in this study only minimal-mild endometriosis patients are placed in the case group and PGP 9.5 is combined with the other markers like VIP (vasoactive intestinal peptide) and SP (subtance P). To test the reliability of the marker we used small intestinal tissue on every slide as positive control. 

On every slide we observed staining in the muscle layers of intestinal tissue, whereas we could demostrate this staining neither on endometriosis nor on control group endometrium slides. We concluded that, with the markers we used, these fibers cannot be demonstrated in the endometrium. Also, we don’t think there is a problem with the endometrial sampling technique. That’s because, even endometrium tissues taken from hysterectomy specimens did not show staining. Endometrium samples taken from hysterectomy specimens are in full thickness and not fragmented which is regarded as gold standart for evaluation.

In the literature, there is one study conducted by Zhang *et al* from China that partially supports our results (15). In this study in both endometriosis and control groups, none of the endometrium samples were stained with NF. But also in the same study, patients with pain symptoms showed significantly more staining with PGP 9.5. However, patients without pain symptoms did not show this difference in both control and endometriosis groups. 

Even on patients with chronic pelvic pain, we couldn’t demonstrate this staining. From this point, we thought that, there might be other factors contributing to staining with PGP 9.5. None of the studies showed difference in staining in different menstruel cycle phases, also. So for now, in order to diagnose endometriosis with endometrial nerve fibers, studies with more patients, preferably divided into subgroups of different symptoms and findings are needed. 

Opposite to Tokushige *et al* study results, we couldn’t detect nerve fibers in the endometrium, and this makes us think that these processes have a more complex infrastructure and many factors might be playing role in this process. Expression is also effected in other cells like dendritic cells. Recently, in a study done by Schulke *et al*, endometriosis patients have significantly more CD1a immature dendritic cells and less CD83 mature dendritic cells in their endometrium in the proliferative phase compared to controls (16). 

This finding could indicate that, just like in cell damage, in endometriosis, monocytes differentiate in a certain direction (17). Since now, in three studies comparing the macrophage cell count in the endometrium of endometriosis patients and healthy women, different results were reported. One of these studies was done by Braun *et al* (18). In their study, all samples were histologically staged after H&E staining and less number of macrophages was detected in the proliferative phase. 

No difference was observed in other menstruel phases. Second study was done by Khan *et al* (19), which shows that there was a significant increase in the number of macrophage cells both in the proliferative and secretory phases compared to control group. Third study was done by Berbic *et al* (20). They reported that there was an increased number of macrophages in the proliferative phase compared to control group. But no comparison was done for secretory phase. In our study we observed an increased number of macrophages only in the proliferative phase (p=0.03). No significant difference was observed in other phases, both in endometriosis and control groups. 

As for proliferative phase, our study showed similar results with Khan *et al* and Berbic *et al* studies. But our study did not show the significant difference in the secretory phase as in Khan *et al* study (p=0.76). Even though we divided the secretory phase into three sub-groups as early, mid and late secretory, we did not find significant difference. 

Although infertility was the only operation indication for first two studies, our study and Berbic *et al* studies included various indications. It’s known that endometriosis causes not only infertility but also chronic pelvic pain, ovarian cysts (endometrioma) and can even be asymptomatic. So, in this kind of studies we think various indications should be included to increase accuracy.

Another variable in studies is the difference of CD68 clones used in the immunohistochemistry. Braun and Berbic have used CD68-PGM1 clone, whereas Khan and us used CD68-KP1 clone. The difference in CD68 cell count may be attributed to this different clones. Immune system cells are difficult to detect immunohistochemically. Even though CD68 is a panmacrophage marker, evidences show that different polarization potentials activates different macrophages (21). 

Due to this, it seems meaningless to compare different anticore clones. Also, inter-laboratory variances in tissue fixation techniques may cause different number of cells stained (22). One of the basic difficulties that has to be dealt with in this kind of studies, is finding the adequate number of patients in each menstruel phase. Also, difficulties determining the exact date of the cycle, might be effecting the results. Maybe the reason of the difference is because of this natural alteration (20). Another self-criticism of the method is the technical difficulties in immunohistochemical staining, which complicate the correct counting of cells and lead to the need for restaining. When it is thought that stage 3-4 endometriosis is a more invasive and adhesive disease, immune system can be expected to be more active in these patients. Due to this, an increased number of macrophages can be expected in these patients.

But in our study we did not find significant difference between stages (p=0.52). The reason for this can be the less number of patients in stages 1-2 or the severity of the immune response seen in the peritoneum is not reflected to the endometrium. Endometriosis is a complex disease with unknown etiology. Because of the point that endometriosis has close relationship with the immune system, this study aimed to investigate a minimal invasive diagnostic method looking for immune responses in the endometrium. 

Based on our results, diagnosis of endometriosis using endometrial samples seems early for now. More studies about this kind of minimal invasive diagnostic methods are needed for this prevalent and suffering disease. 
